# Integrative clinical and biopathology analyses to understand the clinical heterogeneity of infantile rhabdomyosarcoma: A report from the French MMT committee

**DOI:** 10.1002/cam4.2713

**Published:** 2020-02-22

**Authors:** Thibault Butel, Marie Karanian, Gaelle Pierron, Daniel Orbach, Dominique Ranchere, Nathalie Cozic, Louise Galmiche, Aurore Coulomb, Nadège Corradini, Brigitte Lacour, Stéphanie Proust, Florent Guerin, Hélène Boutroux, Angélique Rome, Ludovic Mansuy, Cécile Vérité, Anne‐Sophie Defachelles, Franck Tirode, Veronique Minard‐Colin

**Affiliations:** ^1^ Department of Pediatric and Adolescent Oncology Gustave Roussy (GR) Villejuif France; ^2^ Department of Biopathology and Univ Lyon Université Claude Bernard Lyon 1 INSERM 1052 CNRS 5286 Cancer Research Center of Lyon Lyon France; ^3^ Department of Translational Research and Innovation Centre Léon Bérard Lyon France; ^4^ Department of Molecular Biology Institut Curie Paris France; ^5^ Institut Curie SIREDO Oncology Center (Care, Innovation and research for children and AYA with cancer) PSL Research University Paris France; ^6^ Department of Biostatistics Gustave Roussy (GR) Villejuif France; ^7^ Department of Biopatholgy Necker Hospital Paris France; ^8^ Department of Biopathology Trousseau Hospital Paris France; ^9^ Department of Pediatric and Adolescent Oncology Centre Leon Berard Lyon France; ^10^ French National Registry of Childhood Solid Tumors CHU Nancy France; ^11^ CRESS UMRS1153 INSERM Université Paris‐Descartes Paris France; ^12^ Department of Pediatric and Adolescent Hematogy and Oncology CHU Angers Angers France; ^13^ Department of Pediatric Surgery CHU Bicetre AP‐HP Le Kremlin‐Bicêtre France; ^14^ Department of Pediatric and Adolescent Hematogy and Oncology Trousseau Hospital (AP‐HP) Paris France; ^15^ Department of Pediatric and Adolescent Hematogy and Oncology La Timone Hospital Marseille France; ^16^ Department of Pediatric and Adolescent Hematogy and Oncology Nancy Hospital Nancy France; ^17^ Department of Pediatric and Adolescent Hematogy and Oncology Pellegrin Hospital Bordeaux France; ^18^ Department of Pediatric and Adolescent Oncology Oscar Lambret Centre Lille France; ^19^ Department of Translational Research and Innovation Centre Léon Bérard Univ Lyon Université Claude Bernard Lyon 1 INSERM 1052 CNRS 5286 Cancer Research Center of Lyon Lyon France

**Keywords:** infants, newborns, rhabdoid tumor, rhabdomyosarcoma, spindle cell rhabdomyosarcoma, VGLL2

## Abstract

**Background:**

Rhabdomyosarcoma (RMS) in infants is a particular entity with various clinical presentations and outcomes. To better understand the clinical heterogeneity of RMS in infants, an integrative clinical, histological, and molecular analysis was performed.

**Methods:**

From 1989 to 2015, 37 infants aged less than 6 months with a diagnosis of RMS and archival tumor materials were identified in France. Clinical data, central pathologic review, and molecular profile including RNA sequencing were analyzed.

**Results:**

Nineteen patients (51%) had embryonal RMS (ERMS) (including three highly differentiated ERMS with *PTCH* deletion), eight (22%) had spindle cell RMS (SRMS) (three *VGLL2*‐, one *NTRK*‐, and two (*B)RAF*‐fusions), six (16%) had alveolar RMS (ARMS) (all *FOXO1*‐ or *PAX3*‐fusion), two had unclassified RMS, and two poorly differentiated RMS were retrospectively diagnosed as rhabdoid tumors (RT) with loss of INI1 expression. The two RT patients died of rapid disease progression. Five‐year event‐free (EFS) and overall survival (OS) for RMS were 62% (95%CI, 47‐82) and 52% (95%CI, 37‐72). Eleven patients (31%) relapsed and four (11%) had primary refractory disease (all ERMS). In univariate analysis, EFS and OS were only associated with histology subtype, with 100% survival of known fusion‐positive SRMS. RNA cluster expression showed three main clusters: ARMS, ERMS, and “VGLL2‐fusion” cluster, consisting of SRMS and ERMS.

**Conclusions:**

Biopathology findings from this study support the different prognosis of infantile RMS. New fusion‐positive SRMS has a very good outcome which may allow more conservative treatment in the future.

## Graphical abstract

This study reported integrative clinical, histological, and molecular analysis of infants with RMS. Biopathology findings support the different prognosis of infantile RMS with new fusion‐positive spindle cell RMS having a very good outcome which may allow more conservative treatment in the future.

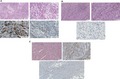

## 1 INTRODUCTION

Rhabdomyosarcoma (RMS) comprises one‐third of soft tissue sarcoma occurring in the first year of life and 5%‐8% of all malignant tumor at this age.^1^ Major subtypes of RMS include alveolar (ARMS) and embryonal (ERMS) tumors. Rarely some other forms are encountered, like spindle cell RMS (SRMS) or sclerosing RMS (ScRMS), often regarded as atypical embryonic forms. SRMS is a poorly defined and heterogeneous morphologic category that seems to be overrepresented in congenital presentations, involves mostly the paratesticular and the head and neck region, and may be associated with a more favorable prognosis.^2^, ^3^, ^4^, ^5^ Furthermore, a rare myogenic transcription factor *MYOD1* mutation has been identified in a subset of aggressive ScRMS, occurring mostly in old children and adults.^6^, ^7^

RMS in newborns and infants, ~4%‐10% of pediatric RMS, is a particular entity with specific clinical presentation and outcome.^1^, ^8^, ^9^, ^10^, ^11^, ^12^ It represents a fascinating and difficult medical challenge because: (a) an important heterogeneity within neonatal RMS presentations has been observed, some tumors being very aggressive and resistant to chemotherapy while others are chemosensitive and easily cured, whereas no diagnosis tools existed to distinguish these entities, and (b) the physiologic immaturity of various organs in infants is responsible for the different metabolism of drugs compared to older patients and potential vulnerability to acute and late effects of therapy, particularly radiotherapy and alkylating agent.^13^, ^14^, ^15^ Recently, molecular rearrangement involving *NCOA2* or *VGLL2* genes have been described in 11 infants with SRMS,^4^, ^7^, ^16^ whereas none of the 30 older children with S/ScRMS were positive for this rearrangements but 10 for *MYOD1* mutation. These *NCOA2‐* or *VGLL2*‐fusion associated RMS seemed to present more favorable outcomes, with no metastatic spread, although limited numbers of cases and only short follow‐up have been reported.

To evaluate the prognosis value of histologic and genetic features in newborns/infants with RMS with modern tools and to correlate with clinical patterns, we reviewed infants with RMS with archival tumor material and treated in France during a 25 years period.

## 2 PATIENTS AND METHODS

### 2.1 Selection criteria

Our retrospective study included all French infants with RMS aged less than 6 months at diagnosis, with tumor tissue available, and prospectively registered in SIOP and European paediatric Soft tissue sarcoma Study Group (EpSSG) studies or recorded in the French National Cancer Registry (FNCR) from January 1989 to June 2015. An age cutoff of 6 months was chosen to focus on the specificity of congenital tumors and avoid analysis of high‐risk RMS patients older than 6 months randomized for chemotherapy induction and maintenance in the EpSSG RMS2005 trial. In addition to the primary cohort from 1989 to 2015, we identified five cases of SRMS with VGLL2‐type fusions diagnosed more recently from 2015 to 2018. Because these cases have less mature follow‐up and represent a common molecular subtype, we present their clinical and outcome data in the on‐line supplement separately from the 1989‐2015 cohort.

### 2.2 Clinical data

Analyses were performed on the data derived from three studies: SIOP malignant mesenchymal tumor (MMT)89 (from 1989 to 1995, n = 6), MMT95 (1995 to 2003, n = 7), and EpSSG RMS2005 (2005 to 2015, n = 12). For patients registered in the FNCR (n = 12) and not included in these studies, data have been extracted from medical files. Clinical staging was defined according to the TNM system and postoperative staging according to the Intergroup Rhabdomyosarcoma Study group.^17^, ^18^ Tumor response evaluation methods are detailed in supporting files.

### 2.3 Treatment

Treatment protocols, SIOP MMT 89,^19^ SIOP MMT 95,^20^ and EpSSG RMS 2005,^21^ have been previously reported. As infants less than 6 months old were not randomized but only registered in these studies, we observed heterogeneous and individually tailored treatments. Radiotherapy was avoided and if necessary, brachytherapy was preferred.

The French MMT committee recommended chemotherapy dose adaptations in the MMT95 and RMS 2005 protocols for infants with RMS aged less than 1 year (see supplementary Table A). Drug dose adaptation was analyzed regarding the current French recommendations in the RMS 2005 and the proportion of patients who received less or more than the recommended doses was determined according to three modalities: “underdosage” (dose at least 25% lower than recommendations for one drug or more, vincristine excepted), “overdosage” (dose at least 25% upper than recommendations for one drug or more, vincristine excepted), and adequate age‐adapted dosage.

### 2.4 Histological data

A centralized histological review of all original slides was performed by two specialized pathologists (DR and MK) to confirm the histological diagnosis and to search for any distinguishing histological features. Diagnosis of RMS was based on morphology and both desmin and myogenin positivity on immunohistochemistry (IHC). RMS were further classified, according to WHO 2013 for tumors of soft tissue,^22^ as ERMS (classical or botryoid), ARMS (with or without confirmation by molecular data), and SRMS. When a tumor did not show the characteristics to be classified in one of these three groups, it was labeled “NOS RMS”. When needed, additional staining including MyoD1, AP2B, and INI was performed.

### 2.5 Molecular data

Array Comparative Genomic Hybridization (CGH array) and Fluorescence In Situ Hybridization (FISH) were performed on paraffin‐fixed tissue. Multiplex reverse transcription polymerase chain reaction (RT‐PCR) assay for the most common sarcoma translocations for routine molecular diagnostic practice was performed on frozen sections when available. Depending on tumor tissue availability, next‐generation RNA sequencing was performed on paraffin and/or on fresh frozen (FF) tissue. RNA sequencing methods are detailed in supporting files.

### 2.6 Statistical considerations

Survival was calculated from the date of the start of treatment to the time of the last follow‐up or death. Event‐free survival (EFS) was calculated from the date of the start of treatment to the date of first event, such as progression, relapse, second malignancy, or death from any cause. Local control was defined as disappearance of all clinical and radiological signs of disease or as stable residual radiographic images for 6 months after completion of treatment. Additional statistical analysis is detailed in supporting files.

### 2.7 Ethical

The study was approved by the Institutional Review Board of Gustave Roussy and the French *Comité de Protection des Personnes Ile‐de‐France Paris VII‐Bicêtre*.

## 3 RESULTS

### 3.1 Patient characteristics

A total of 37 infants were included in the analysis. Thirteen additional patients were screened but excluded because no tumor sample was available for analysis (n = 6) or because the diagnosis of RMS has not been confirmed by histological review (with different morphologic appearance and/or desmin and myogenin negative—n = 7). Thirty‐one cases had adequate tumor material for molecular analysis, either frozen tissue (n = 13), or paraffin‐fixed tissue (n = 9), or both (n = 9). Clinical characteristics at diagnosis are summarized in Table 1. The two main primary locations were non‐parameningeal head and neck (30%) and bladder/prostate (30%). Six tumors (16%) were metastatic at diagnosis, two of which were revised to malignant rhabdoid tumor (MRT) after pathology review.

**Table 1:** Patient characteristics

Characteristics	Number of patients (%)
Total	37 (100)
Age (months)	
Median (range)	2.8 (0‐5.8)
<1 month	10 (27)
1‐3 months	8 (22)
3‐6 months	19 (51)
Sex (boys)	22 (59)
Initial site	
HN non‐PM	11 (30)
GU BP	11 (30)
Limbs	4 (11)
HN PM	3 (8)
GU non‐BP	2 (5)
Other sites	6 (16)
Tumor size > 5 cm	18 (49)
T status	
T1	13 (35)
T2	24 (65)
Lymph Node extension	
N0	31 (84)
N1	6 (16)
IRS stage	
I	3 (9)
II	2 (5)
III	26 (70)
IV	6 (16)
Pathology review	
RMS	35 (95)
Embryonal	19 (54)
Classic	13
Highly differentiated	3
Botryoid variant	2
Anaplastic	1
Spindle cell	8 (24)
Fibrosarcoma‐like	4
VGLL2‐type	3
Triton‐like	1
Alveolar	6 (17)
Not otherwise specified	2 (5)
Rhabdoid tumor	2 (5)

Abbreviations: BP, Bladder‐Prostate; GU non‐BP, Genitourinary non‐Bladder‐Prostate; HN non‐PM, Head and Neck non‐Parameningeal; RMS, rhabdomyosarcoma; T1, Tumor localized to the organ or tissue of origin; T2, Tumor extending beyond the tissue of origin to involve one or more adjacent tissues

### 3.2 Pathology review

**Figure 1:**
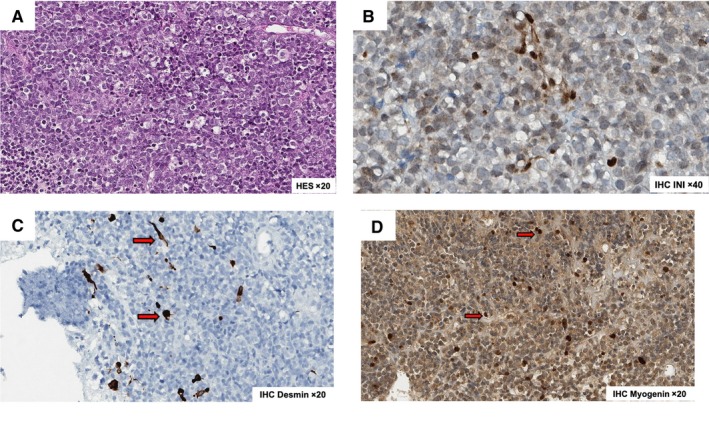
Morphology and immunohistochemistry (IHC) of two rhabdoid tumors initially diagnosed as embryonal rhabdomyosarcoma. A, Hematoxylin‐eosin‐safran [HES] Coloration zoom ×20. B, Loss of nuclear expression of *SMARCB1/INI1* on rhabdoïd cells (note the positive blue staining by endothelial and inflammatory cells). C, Positive immunostaining for desmin (red arrow). D, Positive immunostaining for myogenin (red arrow)

After pathology review of the 37 cases, 19 (51%) were ERMS, eight (22%) were SRMS, six (16%) were ARMS, and two remained NOS RMS (Table 1). Two additional poorly differentiated tumors, initially diagnosed as ERMS (positive for both desmin and myogenin), were reviewed as MRT with SMARCB1/INI1 loss on IHC and confirmed *SMARCB1*/*INI1* mutation on RNAseq (Figure 1).

**Figure 2:**
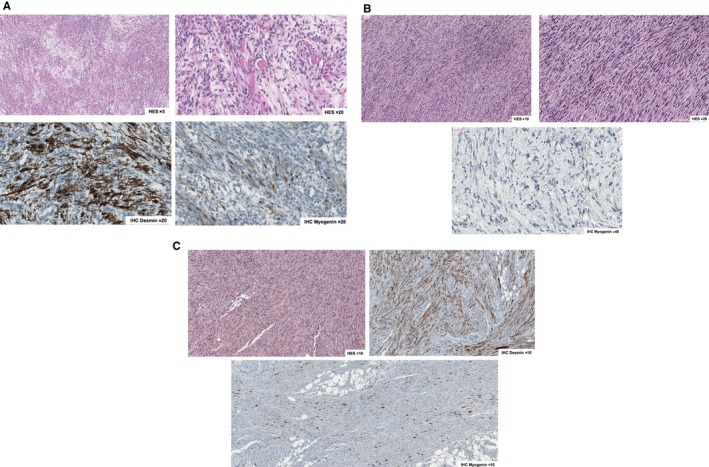
Morphology and IHC of representing infantile rhabdomyosarcoma. A, Highly differentiated embryonal rhabdomyosarcoma. Hematoxylin‐eosin‐safran [HES] coloration zoom x5; Hematoxylin‐eosinsafran [HES] coloration zoom x20; Positive immunostaining for desmin; Positive immunostaining for myogenin (30%). B, VGLL2‐type spindle cell rhabdmyosarcoma. Hematoxylin‐eosin‐safran [HES] coloration zoom x10; Hematoxylineosin‐safran [HES] coloration zoom x20; Positive immunostaining for myogenin (2%). C, “Fibrosarcoma‐like” spindle cell rhabdomyosarcoma. Hematoxylin‐eosin‐safran [HES] coloration zoom x10; Positive immunostaining for desmin; Positive immunostaining for myogenin (5% to 30%)

In the group of 19 ERMS, 13 were labeled classical ERMS, two botryoid ERMS, and one anaplastic ERMS.^22^ Three remaining “rhabdomyoma‐like” cases showed high levels of skeletal muscle differentiation and myoblasts with ample eosinophilic, fibrillary cytoplasm were seen throughout tumor tissue. Marked cellular atypia, as evidenced by nuclear size/polymorphism, hyperchromasia, relatively high mitosis index, and infiltrated margins, supported final RMS diagnosis while rhabdomyoma histology was excluded (Figure 2A).

Among the group of eight SRMS, three cases showed a similar “fibromatous‐like” aspect with few tumor cells on an abundant sclerosing stroma (Figure 2B). The cells were small with moderate atypia and very few mitoses. These easily recognized tumors were suspected to harbor a *VGLL2* rearrangement. Four additional tumors were composed of spindle cells arranged in a fascicular pattern showing a “fibrosarcoma‐like” aspect. In a last patient with SRMS, a 4‐month‐old boy with neurofibromatosis type 1 and bladder/prostate tumor, both diagnoses of SRMS and malignant peripheral nerve sheath tumor (MPNST) with rhabdomyoblastic differentiation (Triton tumor) were considered. It showed atypical histologic presentation similar to adult‐type spindle cell sarcoma consisting of spindle cells with rhabdomyoblastic differentiation in a fascicular architecture and cytonuclear atypia, focal necrosis with high proliferation activity, and heterogeneous staining with desmin, myogenin, and focal staining with proteinS100 without Sox10 expression (Figure S1).

### 3.3 Molecular characterization

RNAseq was performed for all 31 tumor samples with material available for molecular analysis. Additional RT‐PCR was performed for 20/31 patients and CGH array for 10/31 patients. Eleven out of the 31 cases (35%) had fusion genes, including nine cases with known gene fusion (Table 2). All except one ARMS had *PAX3*‐*FOXO1* fusion. One additional *FOXO1‐*negative ARMS had *PAX3*‐positive rearrangement on FISH but no gene fusion was diagnosed on RNAseq. Among the eight SRMS, all three “fibromatous‐like” SRMS had *VGLL2*‐related fusion, with *NCOA2* (n = 2) or *CITED2* partner (n = 1). Three “fibrosarcoma‐like” SRMS presented three rearranged genes previously described in other sarcomas: *TPM3*‐*NTRK*1, *SYPL1*‐*BRAF*,* and TOP2B‐RAF1*. One additional “fibrosarcoma‐like” SRMS had no gene fusion on RNAseq on formalin‐fixed paraffin‐embedded (FFPE) material but no RNAseq was performed on frozen sample (low RNA quality). The last “triton‐like” bladder/prostate SRMS had an unknown fusion *PPHNL1*‐*BEST3*. Two out the three “rhabdomyoma‐like” ERMS had *PTCH1* deletion on CGH array, including one with unknown *BANZ1*‐*FANCC* fusion. No other gene fusion or abnormal CGH was observed in ERMS. No myoD1 mutation was identified in the whole infant RMS cohort.

**Table 2:** Clinicopathologic, molecular features, therapy, and outcome of fusion‐positive infantile RMS

Age (m.)	Primary site	Pathology review (subtype)	Fusion gene (CGH abnormalities)	Status (FU, m.)	Chemotherapy	Surgery	RT (type)
3	Retro‐auricular	ERMS (highly differentiated)	BANZ1B‐FANCC (PTCH1 deletion)	CR1 (58)	VA	Yes (conservative R0)	No
0	Paravertebral	SRMS (VGLL2‐type)	VGLL2‐NCOA2	CR1 (47)	2 Vincristine Cyclo + 4 Vinblastine	Yes (conservative R2)	No
1	Cervical	SRMS (VGLL2‐type)	VGLL2‐CITED2	CR1 (61)	8 VAC/IVA	Yes (conservative R1)	No
1	Forearm	SRMS (VGLL2‐type)	VGLL2‐NCOA2	CR1 (24)	15 VAC	Yes (conservative R2)	No
0	Thigh	SRMS (Fibrosarcoma‐like)	SYPL1‐BRAF	CR1 (65)	2 VA + 7 VAC/VAC	Yes (conservative R0)	No
1	Cheek	SRMS (Fibrosarcoma‐like)	TPM3‐NTRK1	CR1 (138)	3 VA + 4 VAC + 2 VEC	Yes (radical R0)	No
4	Bladder‐prostate	SRMS (Triton‐like)	PPHLN1‐BEST3	Dead	6 IVA	Yes (conservative R0)	No
2	Vagina	SRMS (Fibrosarcoma‐like)	TOP2B‐RAF1	CR2 (65)	7 doxo‐based courses	Yes (conservative R1)	Yes (BT)
4	Retro‐auricular	ARMS	PAX3‐FOXO1	Dead	9 IVA	Yes (conservative R0)	No
4	Nasolabial fold	ARMS	PAX3‐FOXO1	Dead	6 doxo‐based courses	Yes (conservative R2)	Yes (BT)
4	Nasolabial fold	ARMS	PAX3‐FOXO1	Dead	12 doxo‐based courses	Yes (conservative R0)	No

Abbreviations: ARMS, alveolar rhabdomyosarcoma; BT, brachytherapy; CGH, Comparative Genomic Hybridization; CR, complete remission; ERMS, embryonal rhabdomyosarcoma; FU, follow‐up; IVA, vincristine actinomycin‐D ifosfamide; M, months; R0, complete resection; R1, incomplete microscopic resection; R2, incomplete macroscopic resection; SRMS, spindle cell rhabdomyosarcoma; VA, vincristine actinomycin‐D; VAC, vincristine actinomycin‐D cyclophosphamide; VEC, vincristine etoposide cyclophosphamide.

### 3.4 Unsupervised expression analysis

The 22 RMS frozen samples and the 18 FFPE sample were expression profiled (for nine tumors both FFPE specimen and frozen sample were sequenced) but analyzed separately because of technical differences. Except for one SRMS with *VGLL2* fusion identified on frozen sample and not in FFPE sample, there were no discrepancies in results from the analysis of FFPE and frozen specimens. Two samples in the frozen series and four in the FFPE series were not included in the expression analysis because of low RNA yield and poor sequencing quality.

**Figure 3:**
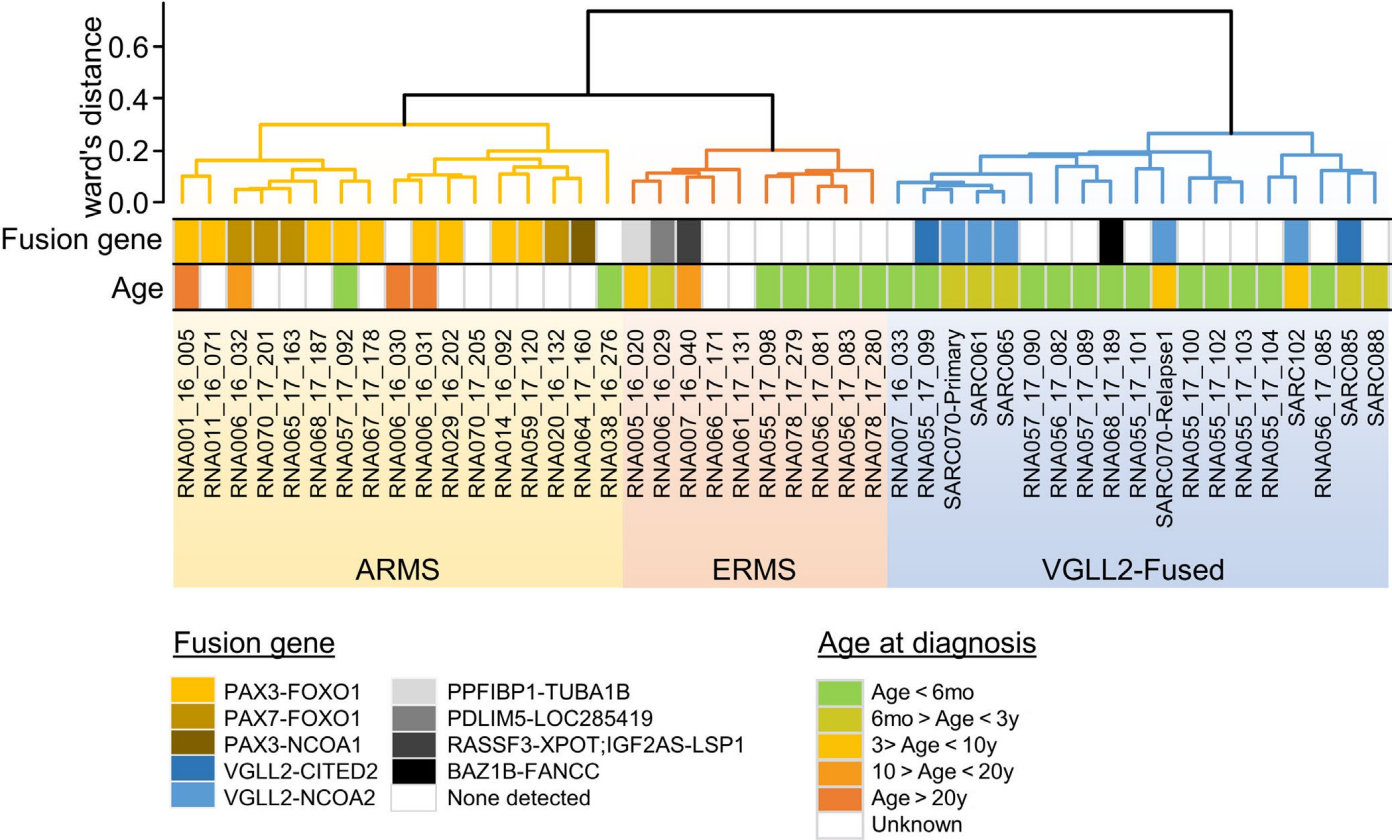
Gene expression clusters using unsupervised consensus hierarchical clustering. For comprehensive and didactical reasons, we mixed our samples with 26 other rhabdomyosarcoma samples of all age combined

First, to confirm the correlation between histology and sample gene expression profiles, we analyzed the 20 RMS frozen sample cases with 184 others soft tissue sarcoma of all age combined.^23^ All 20 cases were grouped in RMS subgroups except the MRT case. To identify the potential expression subgroups consensus in the RMS group, we focused on frozen samples only, combining the 19 samples with 26 RMS samples of all age confounded (Figure 3). Cluster expression analysis showed that three‐cluster model best fits the data when several clustering conditions were applied. The group 1 was composed of the ARMS (2/19, 10%), the group 2 contained ERMS (5/19, 26%), the group 3, the “VGLL2‐fusion” cluster, consisted of all six SRMS and six ERMS (including the two RMS with *PTCH1* deletion).

### 3.5 Treatment

The two patients with metastatic MRT diagnosed after pathology review died of rapid disease progression despite intensive therapy and were not further included in the therapy and outcome analysis. All patients received chemotherapy. Nineteen patients (54%) received IVA/VAC chemotherapies, three (9%) were treated with VA only (one died of toxicity after the first course of VA chemotherapy), and 13 (37%) had anthracycline‐based or other intensive regimens either because of insufficient response to IVA/VAC (n = 7), metastatic disease at diagnosis (n = 3), lymph node involvement (n = 1), or two by physician decision. Twenty‐eight patients had surgery as part of their first‐line therapy, including 21 after chemotherapy. Ten patients had incomplete microscopic (n = 8) or macroscopic (n = 2) secondary surgery: four had additional brachytherapy (one ARMS had nodal relapse) while the other six did not have additional local therapy: three had local relapse (one ARMS, one classic ERMS, and one RMS NOS) and three remain in CR1 (two VGLL2‐SRMS and one highly differentiated ERMS). In total, four infants (11%) have been irradiated during first‐line therapy, all with brachytherapy.

### 3.6 Outcome and prognostic factors

**Figure 4:**
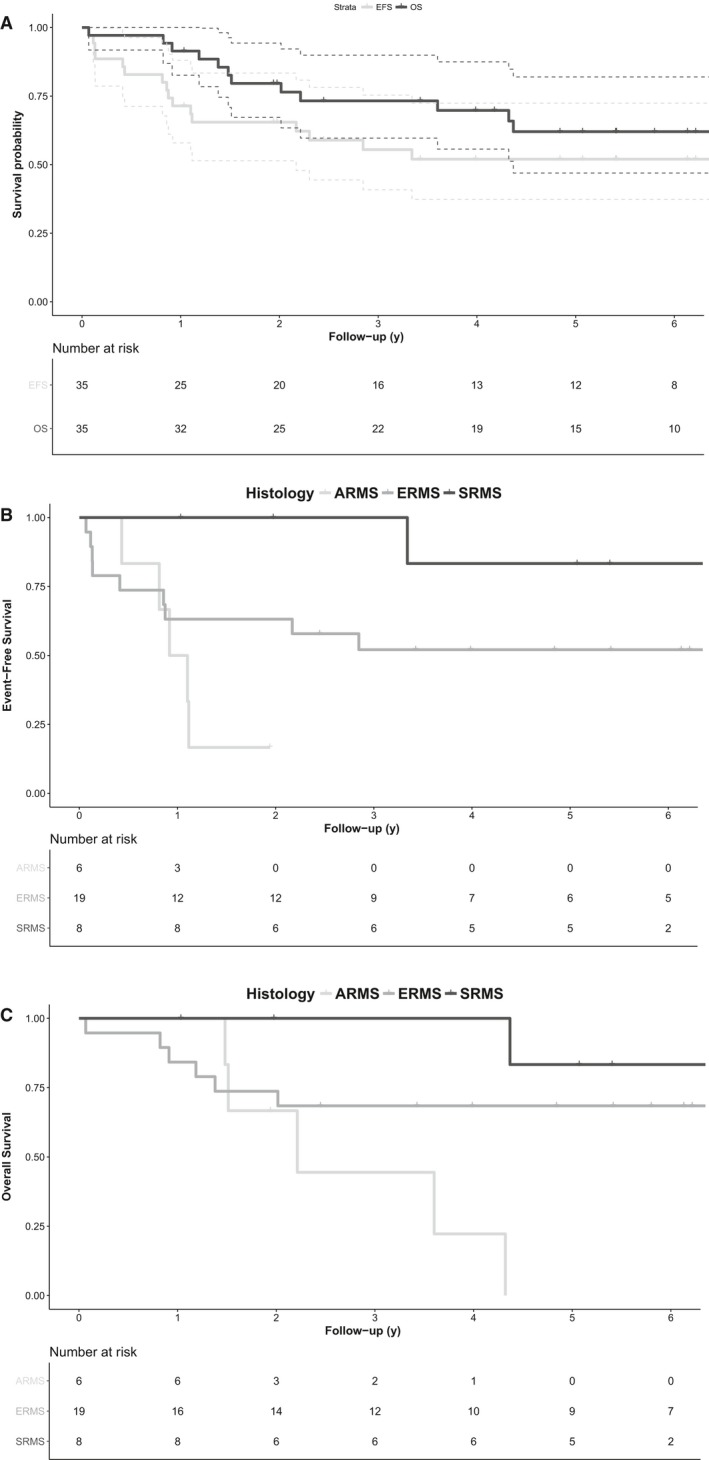
Five‐year overall (OS) and event‐free survivals (EFS) or the whole population. Five‐year EFS according to histology subtype. Five‐year OS according to histology subtype

At cutoff date, the mean follow‐up of survivors was 6.3 years (range 1‐13 years). Five‐year overall survival (OS) and EFS rates for all 35 patients were 62% (95%CI, 47‐82) and 52% (95%CI, 37‐72) (Figure 4). The 5‐year OS and EFS rate for patients with localized RMS were, respectively, 65% (95%CI, 49‐85) and 56% (95%CI, 41‐78). Sixteen infants had events. Eleven patients relapsed (seven deaths), mostly on primary site (n = 6) or regional lymph nodes (n = 3) but also metastatic (n = 2). The median time from diagnosis to relapse was 13 months (range, 5‐40). Four patients (11%, all classic ERMS) had primary refractory disease and died of disease progression (supplementary Table B). One 5‐week‐old boy died of treatment‐related toxicity (see below). In total, 23 patients are alive, of whom 19 in first remission and four in subsequent remission after local relapse. Analysis of 5‐year EFS and OS rates by prognostic variables is shown in supplementary Table B. In this small sample size, OS and EFS differed by histology status only, with ARMS having the worst outcome and SRMS achieving the best survivals (Figure 4B,C). Indeed, all except one SRMS (“triton‐like” SRMS) are alive in CR1.

### 3.7 Chemotherapy toxicity

Doses were escalated successfully for the majority of infants. Half of patients received weight‐adapted chemotherapy dose according to national recommendations, while 34% were overdosed and 9% were underdosed. Main chemotherapy toxicities are shown in Supplementary Table C. There was one treatment‐related death, a 5‐week‐old boy, with clinical and biological symptoms of abrupt sinusoidal obstruction symptoms after chemotherapy overdosage (about 250% of the recommended cyclophosphamide and actinomycin‐D doses). Additionally, most frequent grade 3 and 4 toxicities were infections (70%) and three infants (9%), aged 3 weeks, 4 and 5 months, respectively, had grade 3 hepatic veno‐occlusive disease after VAC therapy. Twenty‐five infants (68%) received ifosfamide. One acute grade 3 tubular toxicity with severe hyponatremia was recorded as well as one acute neurologic toxicity without detailed information. Long‐term toxicity was not reviewed.

## 4 DISCUSSION

This study reviewed clinical, histological, and molecular data of 37 RMS in infants younger than 6 months and treated in France over a 25 years period. SMARCB1/INI1 staining is important in this population with two infants with desmin‐ and myogenin‐positive poorly differentiated metastatic RMS secondarily diagnosed as INI1‐deficient RT, with rapid disease progression and death. Moreover, biopathology findings support the different prognosis of this heterogeneous population with fusion‐positive SRMS having a very good outcome while ARMS but also ERMS having an aggressive course with ~50% of treatment failures.

Spindle cell/sclerosing RMS have been redefined in the WHO 2013 classification as a stand‐alone pathologic entity, distinct from ERMS.^22^ However, the definition criteria of SRMS are not consensual, because “spindle cells” are observed in different types of RMS‐like ERMS, RMS with *VGLL2* rearrangement or with *MyoD1* mutation. This morphological term does not define a precise entity but a heterogeneous group with different tumors. Allagio *et al* reported seven cases of *VGLL2*‐positive RMS which showed a striking resemblance with our cases, with monomorphic tumor cells, delicate and scant eosinophilic cytoplasm, and oval to wavy nuclei.^5^ All tumors presented patchy to diffuse desmin staining, and scattered, multifocal myogenin, and MyoD1 reactivity. Our cohort, along with this published by Allagio and our more recent French experience, confirmed the excellent outcome of VGLL2‐type SRMS. Typically, infants present at very young age—but older infants have been also diagnosed—with localized disease, in the back, but also in the head and neck or limbs, and may have poor tumor response to chemotherapy (in the present cohort, among the seven evaluable SRMS, two had partial response and four had SD/minor response). In this cohort, and in addition to cases diagnosed after June 2015 (Table 2), none of the eight VGLL2‐type SRMS relapsed. Importantly, all infants except one had RMS‐tailored chemotherapy but seven had incomplete surgery and one had no local therapy while none had additional radiotherapy. These results, along with those published by others,^4^, ^7^, ^16^ allow to recommend a more conservative approach for SRMS in the future, especially avoiding mutilating surgery and/or radiotherapy.

Previous publications reported PTCH1‐inactivating mutations (resulting in hedgehog (Hh)‐signaling pathway activation) or a deregulation of the Hh‐signaling pathway in RMS tumors with high levels of myogenic differentiation.^24^, ^25^ As previously suggested by others,^24^ highly differentiated RMS tumors and rhabdomyomas might form a continuous spectrum of tumors. Notably, no infant had nevoid basal cell carcinoma syndrome (or Gorlin syndrome), characterized by a *PTCH1* inactivating mutation.^26^, ^27^ Lastly, three SRMS sharing morphological characteristics with spindle cell sarcoma carry fusions (ie, *BRAF*‐*SYPL1*, *TPM3‐NTRK*, or *TOP2B*‐*RAF1*) reported in other types of MMT including infantile fibrosarcoma.^28^, ^29^, ^30^, ^31^, ^32^, ^33^, ^34^ The nosological status of this subgroup remained uncertain. These specific SRMS may represent “a grey zone” between RMS and other MMT and may benefit from adapted treatment in the future including novel targeted therapies.^28^, ^35^, ^36^, ^37^

The current series also confirms the dismal outcome of ERMS and ARMS in infants. Notably, we observed a significant number of early progressions (11%) when compared with RMS in older patients (2%).^38^ All occurred in fusion‐negative non‐highly differentiated ERMS and despite intensive chemotherapy, lead to death. It may suggest a specific biologic *substratum* of some infantile ERMS although it was not demonstrated in the current RNAseq analysis. Importantly, no myoD1 mutation was observed, even in SRMS, confirming that it occurs mainly in older patients.^7^ Recently, the Children's Oncology Group (COG) reported outcome of 124 children aged ≤24 months, who were enrolled on ARST0331 and ARST0531 trials.^39^ No SRMS or highly differentiated ERMS were specifically identified. The 5‐year EFS and OS rates for the whole cohort were 68% and 82%. EFS was significantly higher among patients who were aged 12 to 24 months, and underwent protocol‐specified therapy, including radiotherapy (58% of patients). The 5‐year EFS for infants <1 year was 58% which is slightly better that the EFS of 49% of our series in which four infants only (11%) have been irradiated (all brachytherapy) but 83% underwent surgery, underlying the different treatment strategy among cooperative groups.

Finally, after pathology review and molecular analysis, two poorly differentiated desmin‐ and myogenin‐positive RMS (Figure 1) were diagnosed as MRT with INI1 loss both on IHC and transcriptome analysis. Desmin and myogenin expressions were reported here for the first time in RT. It underscores that INI1 immunostaining is required to allow MRT diagnosis, even in desmin/myogenin‐positive tumor, and should be systematically performed for poorly differentiated RMS in infants. Indeed, the rhabdoid phenotype is a final common morphologic pathway assumed by poorly differentiated tumors with dissimilar patterns of differentiation. Although genetically similar, recent data demonstrated that RTs may be epigenetically very different with potentially different cellular origin(s)^40^ and do not represent a unified group of neoplasms with regard to cellular lineage. Indeed, the rhabdoid phenotype is a final common morphologic pathway assumed by poorly differentiated tumors with dissimilar patterns of differentiation.^40^ Moreover, the clinical phenotype of the two patients (metastatic presentation, primary refractory disease despite intensive therapy, and rapid death), as well as clustering analysis, also support MRT diagnosis.

In conclusion, biopathology findings of our work support the different prognosis of this heterogeneous population of infantile RMS with *VGLL2* rearranged SRMS having a very good outcome while ARMS but also ERMS having a clinically aggressive course. Fusion‐positive SRMS appear to behave as intermediate malignancy tumor and may benefit from more conservative strategies in the future. For ERMS and ARMS, alternative approaches are required, along with larger studies and international collaborations, to investigate underlining biological mechanisms of tumor aggressiveness especially in infantile ERMS.

## CONFLICT OF INTEREST

None.

## AUTHOR CONTRIBUTIONS

All authors have reviewed, discussed, and agreed to their individual contributions prior to submission. Butel T, Karanian M, Pierron G, Ranchère D, Tirode F, and Minard‐Colin V: Conceptualization, formal analysis, investigation, funding acquisition, writing original draft. Orbach D, Galmiche L, Colomb A, Corradini N, Lacour B, Proust S, Guerin F, Boutroux H, Rome A, Mansuy L, Vérité C, Desfachelle AS: conceptualization, data curation. Butel T, Cozic N, and Tirode F: data curation, methodology, formal analysis.
