# Supplementary material for: Integrative clinical and biopathology analyses to understand the clinical heterogeneity of infantile rhabdomyosarcoma: A report from the French MMT committee
Source: Cancer Med. 2020 Feb 22;9(8):2698–709. doi: 10.1002/cam4.2713 (PMC7163108; doi:10.1002/cam4.2713)
Supplement: Supplementary file 1 [file CAM4-9-2698-s001.pdf]

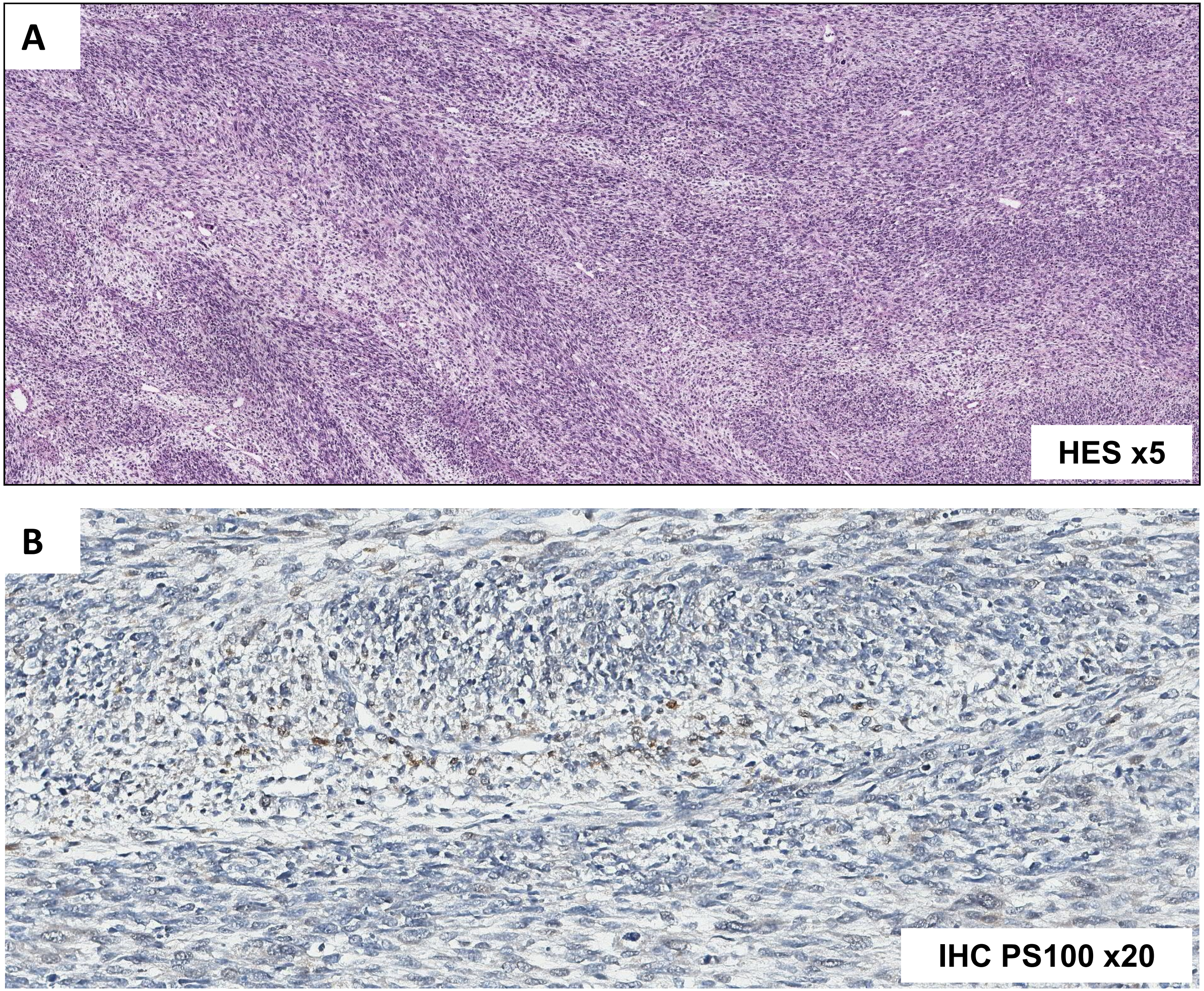

Supplementary Figure 1. **Morphology and Immunohistochemistry (IHC) of « Triton-like » SRMS**
A- Hematoxylin-eosin-safran [HES] Coloration zoom x5 B- Focal Positive immunostaining for PS100
