# Supplementary material for: Integrative clinical and biopathology analyses to understand the clinical heterogeneity of infantile rhabdomyosarcoma: A report from the French MMT committee
Source: Cancer Med. 2020 Feb 22;9(8):2698–709. doi: 10.1002/cam4.2713 (PMC7163108; doi:10.1002/cam4.2713)
Supplement: Supplementary file 2 [file CAM4-9-2698-s002.doc]

**Supplementary Material**

**Clinical Data**

The span of years of diagnosis implied that differences in staging technology have evolved but imaging was not reviewed for the purpose of this study. Tumor response had been evaluated according to volumetric response. Complete response (CR) was a complete disappearance of all visible disease, partial response (PR) was a tumor volume reduction of at least 2/3 of the initial tumor volume, progressive disease (PD) was any increase of more than 40% in volume of any measurable lesion, or appearance of new lesions and a stable disease (SD) was an evolution with no criteria for PR or PD (< 33% tumor volume reduction or increase of less than 40%).

**Molecular data**

Total RNAs were isolated from crushed FF tumors or from FFPE tissue sections using a Trizol reagent kit (Thermo Fisher Scientific, Courtaboeuf, France). All RNA were quantified by spectrophotometry (NanoDrop; Thermo Fisher Scientific) and quality was controlled (DV200 value cutoff > 13%) using a TapeStation with Hs RNA ScreenTape (Agilent Technologies, Santa Clara, CA, USA). Library constructions were performed following the TruSeq Stranded mRNA LS protocol (Illumina, San Diego, CA, USA) for FF samples or TruSeq RNA Access Library Prep Kit (Illumina) for FFPE samples. Sequencing were performed on either HiSeq 2500 (150nt paired-end, FF samples) or NextSeq 500 (75nt paired-end, FFPE samples) Illumina sequencing machines.

Alignments were performed either with TopHat2 (FF^1^) with the Hg19 reference genome or using STAR algorithm (FFPE^2^) with the GRCh38 reference genome. Fusion gene assessments were made using DeFuse^3^, STAR-Fusion^4^, FusionCatcher^5^ and FusionMap tools. Expression profiles were extracted from fastq files using Kallisto^6^, and transformed as log2(TPM+2) prior to quantile normalization using the Limma package v3.32.2 performed in the R environment v3.4.1^7^. Only genes with a coding sequence annotation (based on Ensembl GRCh38p5 annotation) and with a maximum expression value across all samples above 2 were considered for the clustering analyses, which was performed using Ward’s distance on the 10% most variant genes based on their interquartile range.

**Statistical Considerations**

The cut-off date for analyses was June 2017, providing a minimum potential follow-up of 24 months from the last date of study entry. The survival curves were estimated using Kaplan Meier analysis. The statistical analysis of each variable was tested in an univariate analysis using cox models. Survival estimates were presented followed by confident interval (CI95). For toxicity analysis, all 3-5 grade toxicities defined by NCI Common Terminology Criteria for Adverse Events (CTCAE) v4.0 for each patient in all cycles of chemotherapy was used. All analyses were performed with R version 3.2.3.

1. Kim D, Pertea G, Trapnell C, Pimentel H, Kelley R, Salzberg SL. TopHat2: accurate alignment of transcriptomes in the presence of insertions, deletions and gene fusions. Genome Biol. 2013;14(4):R36.

2. Dobin A, Davis CA, Schlesinger F, et al. STAR: ultrafast universal RNA-seq aligner. Bioinforma Oxf Engl. 2013;29(1):15-21.

3. McPherson A, Hormozdiari F, Zayed A, et al. deFuse: an algorithm for gene fusion discovery in tumor RNA-Seq data. PLoS Comput Biol. 2011;7(5):e1001138.

4. Haas B, Dobin A, Stransky N, et al. STAR-Fusion: Fast and Accurate Fusion Transcript Detection from RNA-Seq. bioRxiv. March 2017:120295.

5. Nicorici D, Satalan M, Edgren H, et al. FusionCatcher - a tool for finding somatic fusion genes in paired-end RNA-sequencing data. bioRxiv. November 2014:11650.

6. Bray NL, Pimentel H, Melsted P, Pachter L. Near-optimal probabilistic RNA-seq quantification. Nat Biotechnol. 2016;34(5):525-527.

7. The R Core Team. R: The R Project for Statistical Computing. https://www.r-project.org/. 2016.

**Table A. Current French chemotherapy dose adaptation recommendations for infants (all doses are in mg/kg and for one course)**

|  | **0-3 months**  **or**  **<6 kg** | **3-6 months**  **or**  **6-8 kg** | **> 6 months**  **or**  **> 8 kg** |
| --- | --- | --- | --- |
| **Vincristine** | 0.025 → 0.05# | 0.033 → 0.05# | 0.05 |
| **D Actinomycin** | 0.025 → 0.05# | 0.033 → 0.05# | 0.05 |
| **Ifosfamide** | Contraindicated before 6 months or 8 kg | | 50 → 100 Days 1-2 |
| **Cyclophosphamide** | 25 → 50 | | 30 → 50 |
| **Doxorubin** | Contraindicated before 3 months  or 6 kg | 0.65 → 1 on days 1-2 | 1 on days 1-2 |

 progressive increase according to tolerance

**Table B. Prognostic Variables for Overall Survival (OS) and Event-Free Survival (EFS)**

|  | | | 5y-EFS | | 5y-OS 5y | |
| --- | --- | --- | --- | --- | --- | --- |
|  | | Nb of patients | HR | p | HR | p |
| Sex | Boys | 22 | - | - | - | - |
|  | Girls | 13 | 1.2 | 0.8 | 1.5 | 0.5 |
| Age, in months | < 3 months | 19 | - | - | - | - |
|  | > 3 months | 16 | 1.2 | 0.7 | 1.2 | 0.7 |
| Initial Site | Favorable | 12 | - | - | - | - |
|  | Unfavorable | 23 | 1.3 | 0.6 | 0.8 | 0.7 |
| IRS group | I & II | 5 | - | 0.1 | - | 0.6 |
|  | III | 26 | 2.2 |  | 0.9 |  |
|  | IV | 4 | 6 |  | 2.1 |  |
| T Status | T1 | 13 | - | - | - | - |
|  | T2 | 22 | 1.5 | 0.5 | 0.8 | 0.7 |
| Tumor Size, cm | < 5 cm | 19 | - | - | - | - |
|  | ≥ 5 cm | 16 | 0.4 | 0.08 | 0.6 | 0.4 |
| Metastatic | M0 | 31 | - | - | - | - |
|  | M1 | 4 | 2.8 | 0.1 | 2.2 | 0.31 |
| Lymph node involvement | N0 | 29 | - | - | - | - |
|  | N1 | 6 | 1.6 | 0.4 | 2,4 | 0.2 |
| Histology | Non ARMS | 29 | - | - | - | - |
|  | ARMS | 6 | 3.8 | 0.02 | 4.8 | 0.005 |
| Histology | Non SRMS | 27 | - | - | - | - |
|  | SRMS | 8 | 0.2 | 0.046 | 0.3 | 0.16 |
| Quality of resection | R0 | 19 | - | - | - | - |
|  | R1 & R2 | 8 | 2.7 | 0.1 | 0.9 | 0.9 |

|  | **Whole cohort (n=37)** | **Under-dosed &**  **Well weight-adapted**  **(n=23)** | **Over-dosed**  **(n=12)** |
| --- | --- | --- | --- |
| **Toxicity**  ***Infectious***  ***Digestive***  ***VOD***  ***Neurologic***  ***Cardiac***  ***Pulmonary***  ***Hearing***  ***Renal***  ***Allergic***  ***NA*** | **26 (79%)**  23 (70%)  7 (21%)  3 (9%)  2 (6%)  0  1 (3%)  0  3 (9%)  1 (3%)  4 | **16 (80%)**  14 (70%)  6 (30%)  2 (10%)  1 (5%)  -  1 (5%)  -  2 (10%)  0  3 | **10 (83%)**  9 (75%)  1 (8%)  1 (8%)  1 (8%)  -  0 (0%)  -  1 (8%)  1 (8%)  - |
| **Toxic death** | 1 (3%) | 0 | 1 (8%) |
| **Cumulative number of toxicities** | 2.2 (0-8) | 2.4 (0-8) | 2.2 (0-6) |
| **Total number of infectious toxicities** | 1.8 (0-5) | 1.6 (0-5) | 1.7 (0-5) |
| **Number of toxicities**  **(per course)** | **0.4** | **0.3** | **0.6** |

**Table C. Grade 3/4 toxicity regarding chemotherapy dose adaptation**

VOD, Veno-occlusive disease

**Table D. Clinicopathologic, molecular features, therapy and outcome of additional cases of VGLL2 type SRMS diagnosed from June 2015 to April 2018**

| **Age**  **(m.)** | **Primary**  **site** | **Pathology**  **Review**  **(subtype)** | **Fusion gene (CGH abnormalities)** | **Status**  **(FU, m.)** | **Chemotherapy** | **Surgery** | **RT (type)** |
| --- | --- | --- | --- | --- | --- | --- | --- |
| 8 | Thigh | SRMS  (VGLL2 type) | VGLL2-NCOA2 | CR1  (26) | 9 VAC/IVA + 6 months VC maintenance | Yes  (conservative R1) | No |
| 14 | Paravertebral | SRMS  (VGLL2 type) | VGLL-/CITED2 | CR1  (12) | 9 VAC/IVA + 6 months VC maintenance | No | No |
| 10 | Retro-auricular | SRMS  (VGLL2 type) | VGLL2-CITED2 | CR1  (29) | 9 VAC/IVA | Yes  (conservative R1) | No |
| 3 | Forearm | SRMS  (VGLL2 type) | NCOA2-TEAD1 | CR1  (13) | 9 VAC/IVA + 6 months VC maintenance | Yes  (conservative R1) | No |
| 2 | Forearm | SRMS  (VGLL2 type) | NCOA2-TEAD1 | CR1  (30) | 4 VA +8 IVA | Yes  (conservative R1) | No |

**Figure 1.**
